# Metaproteomics in the One Health framework for unraveling microbial effectors in microbiomes

**DOI:** 10.1186/s40168-025-02119-5

**Published:** 2025-05-23

**Authors:** Robert Heyer, Maximilian Wolf, Dirk Benndorf, Sergio Uzzau, Jana Seifert, Lucia Grenga, Martin Pabst, Heike Schmitt, Bart Mesuere, Tim Van Den Bossche, Sven-Bastiaan Haange, Nico Jehmlich, Mariagrazia Di Luca, Manuel Ferrer, Sergio Serrano-Villar, Jean Armengaud, Helge B. Bode, Patrick Hellwig, Catherine Robbe Masselot, Renaud Léonard, Paul Wilmes

**Affiliations:** 1 Multidimensional Omics Analyses Group, Leibniz-Institut für Analytische Wissenschaften – ISAS – e.V., Bunsen-Kirchhoff-Straße 11, 44139 Dortmund, Germany; 2 Multidimensional Omics Analyses Group, Faculty of Technology, Bielefeld University, Universitätsstraße 25, 33615 Bielefeld, Germany; 3 Bioprocess Engineering, Otto-Von-Guericke University Magdeburg, Universitätsplatz 2, 39106 Magdeburg, Germany; 4 Bioprocess Engineering, Max Planck Institute for Dynamics of Complex Technical Systems Magdeburg, Sandtorstraße 1, 39106 Magdeburg, Germany; 5 Applied Biosciences and Process Engineering, Anhalt University of Applied Sciences, Köthen, Germany; 6 Department of Biomedical Sciences, University of Sassari, 07100 Sassari, Italy; 7 Institute of Animal Science, University of Hohenheim, Emil-Wolff-Str, Stuttgart, Germany; 8 HoLMiR - Hohenheim Center for Livestock Microbiome Research, University of Hohenheim, Leonore-Blosser-Reisen Weg, Stuttgart, Germany; 9 Département Médicaments Et Technologies Pour La Santé (DMTS), Université Paris-Saclay, CEA, INRAE, SPI, Bagnols-Sur-Cèze, France; 10 Department of Biotechnology, Delft University of Technology, Delft, The Netherlands; 11 Institute for Infectious Disease Control, National Institute for Public Health and the Environment (RIVM), Bilthoven, The Netherlands; 12 Department of Applied Mathematics, Computer Science and Statistics, Ghent University, 9000 Ghent, Belgium; 13 VIB - UGent Center for Medical Biotechnology, VIB, 9052 Ghent, Belgium; 14 Department of Biomolecular Medicine, Faculty of Medicine and Health Sciences, Ghent University, 9052 Ghent, Belgium; 15 Department of Molecular Toxicology, Helmholtz-Centre for Environmental Research - UFZ GmbH, Permoserstrasse 15, 04318 Leipzig, Germany; 16 Department of Biology, University of Pisa, 56127 Pisa, Italy; 17 Instituto de Catalisis y Petroleoquimica (ICP), CSIC, 28049 Madrid, Spain; 18 Department of Infectious Diseases, Hospital Universitario Ramon y Cajal, Instituto de Investigación Sanitaria Ramón y Cajal (IRYCIS), CIBER de Enfermedades Infecciosas, Madrid, Spain; 19 Department of Natural Products in Organismic Interactions, Max-Planck-Institut for Terrestrial Microbiology, Karl-Von-Frisch-Str. 10, 35043 Marburg, Germany; 20 Center for Synthetic Microbiology (SYNMIKRO), Phillips University Marburg, 35043 Marburg, Germany; 21 Department of Chemistry, Phillips University Marburg, 35043 Marburg, Germany; 22 Bioprocess Engineering, Max Planck Institute for Dynamics of Complex Technical Systems, Sandtorstraße 1, 39106 Magdeburg, Germany; 23 Université de Lille, CNRS, UMR, 8576 - UGSF Lille, France; 24 Luxembourg Centre for Systems Biomedicine, University of Luxembourg, L-4362 Esch-Sur-Alzette, Luxembourg; 25 Department of Life Sciences and Medicine, University of Luxembourg, L-4362 Esch-Sur-Alzette, Luxembourg

**Keywords:** Microbiome, **Microbial community**, **Metaproteomics**, Bacteriophages, Microbial effectors

## Abstract

One Health seeks to integrate and balance the health of humans, animals, and environmental systems, which are intricately linked through microbiomes. These microbial communities exchange microbes and genes, influencing not only human and animal health but also key environmental, agricultural, and biotechnological processes. Preventing the emergence of pathogens as well as monitoring and controlling the composition of microbiomes through microbial effectors including virulence factors, toxins, antibiotics, non-ribosomal peptides, and viruses holds transformative potential. However, the mechanisms by which these microbial effectors shape microbiomes and their broader functional consequences for host and ecosystem health remain poorly understood. Metaproteomics offers a novel methodological framework as it provides insights into microbial dynamics by quantifying microbial biomass composition, metabolic functions, and detecting effectors like viruses, antimicrobial resistance proteins, and non-ribosomal peptides. Here, we highlight the potential of metaproteomics in elucidating microbial effectors and their impact on microbiomes and discuss their potential for modulating microbiomes to foster desired functions.

**Graphical Abstract:**

Word Cloud showing the abundance of keywords in combination with the “Microbiome” in PubMed NCBI. As abundance values, the rounded logarithm with the base of 2 hits were used and submitted to https://wordart.com/create. For microbiome, the number without any combination was used for calculation. The word cloud displays different aspects of microbiome research: (i.) sources of microbiomes (green), (ii.) interactions (purple), (iii.) involved taxa (red), (iv.) applied experimental approaches (blue), and (vi.) societal effects and recent or future applications (gray).

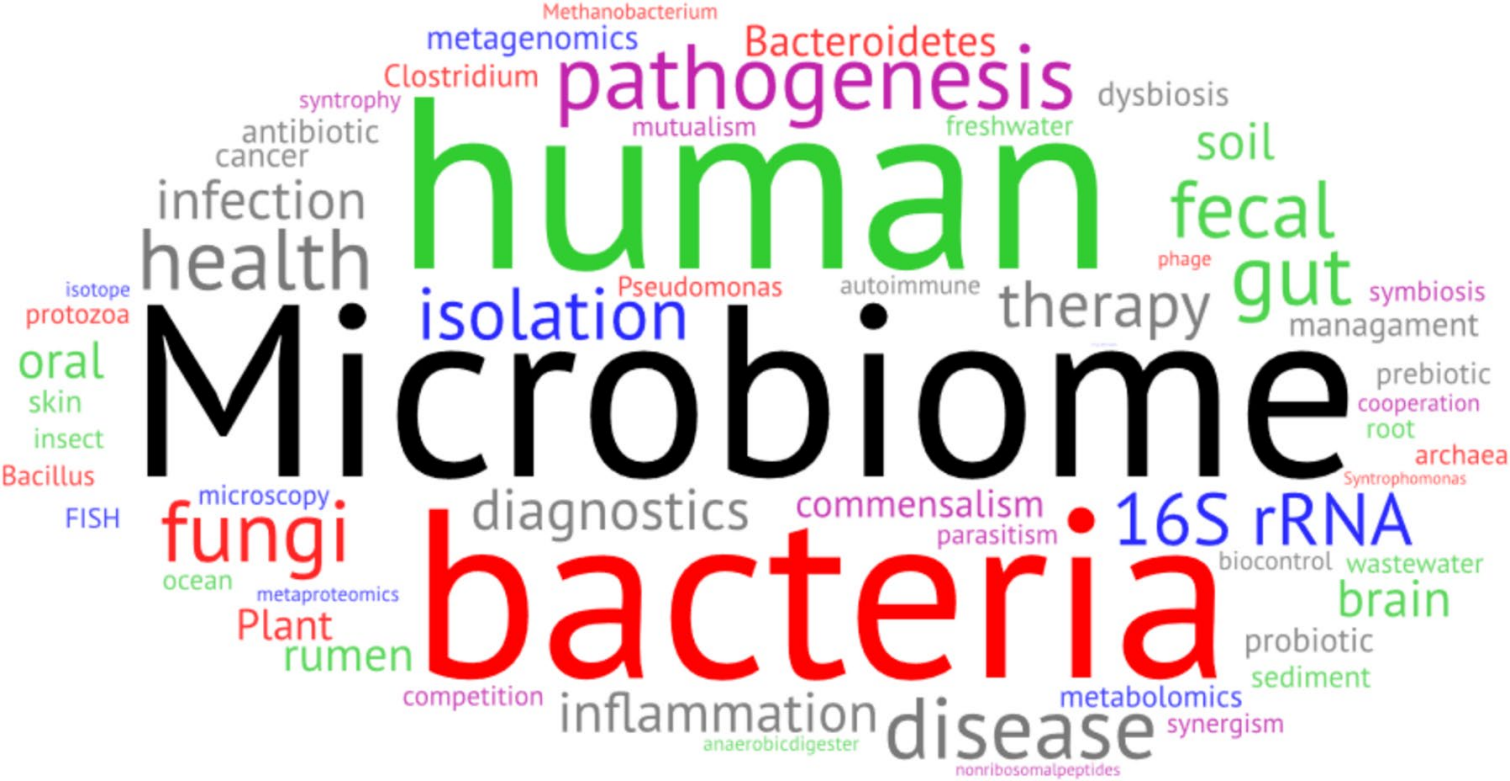

Video Abstract

**Supplementary Information:**

The online version contains supplementary material available at 10.1186/s40168-025-02119-5.

## Introduction

The One Health framework is based on the deep interconnection among human health, animals, and agricultural and environmental ecosystems. These interactions are evident in examples such as zoonotic diseases and the emerging spread of antibiotic resistance. A key factor linking human and animal health with environmental, agricultural, and biotechnological systems is their microbiome. The exchange of cellular organisms, viruses, plasmids, transposons, and genes between different microbiomes leads to (i.) alterations in microbiome composition and function within recipient systems, (ii.) the introduction of pathogenic species and genes, and (iii.) the transfer of antibiotic resistance genes. In relation to the latter, in 2019 alone, deaths related to antimicrobial resistance (AMR) were estimated at 4.71 million worldwide, including 1.14 million deaths attributable to bacterial AMR [1]. Conversely, controlling microbiome composition holds transformative potential for healthcare and biotechnological applications. Identifying microbial effectors, such as virulence factors, toxins, antibiotics, non-ribosomal peptides (NRP)s, and viruses across diverse environments, is crucial for precisely managing microbial communities [2, 3].

While microbiome-based effectors hold significant potential for future applications, a deeper understanding of the mechanisms by which these effectors influence microbiomes and broader functional impacts remains essential.

Metaproteomics provides a powerful toolbox to identify and monitor microbial effectors [4]. It has already proven valuable across diverse applications, including characterizing the impact of antibiotic therapy on human gut microbiomes [5], assessing antibiotic resistance in animals [6] and their manure [7], evaluating the impact of mycotoxins on animal gut microbiomes [8], exploring alternative genes encoded in human gut bacteriophages (phages) [9], characterizing the human gut virome [10], and identifying phage populations within anaerobic digesters [11].

Recent advancements in high-resolution mass spectrometers and overall progress in metaproteomics have significantly improved the sensitivity and specificity of microbial effector identification. Further refinements in the metaproteomic workflow hold great promise for advancing our understanding of microbial effectors and their impact on microbiomes.

In this opinion piece, we discuss how metaproteomics provides insights into the occurrence of microbial effectors and how these influence microbiomes. We focus on seven key challenges: (i.) detecting and quantification of low-abundant microbial effectors, (ii.) identification of non-canonical peptides and proteins (e.g., NRPs), (iii.) using search databases for microbial effectors, (iv.) taxonomic and functional annotation of microbial effectors, (v.) mapping of microbial effectors to their hosts and targets, and (vi.) identification strategies to explore their interactions. To address these questions, we will first introduce the microbial effectors, then outline the metaproteomics workflow and the required adaptations for microbial effector analysis, before focusing on their application in microbiomes within the framework of One Health.

## Microbial effectors

**Fig. 1:**
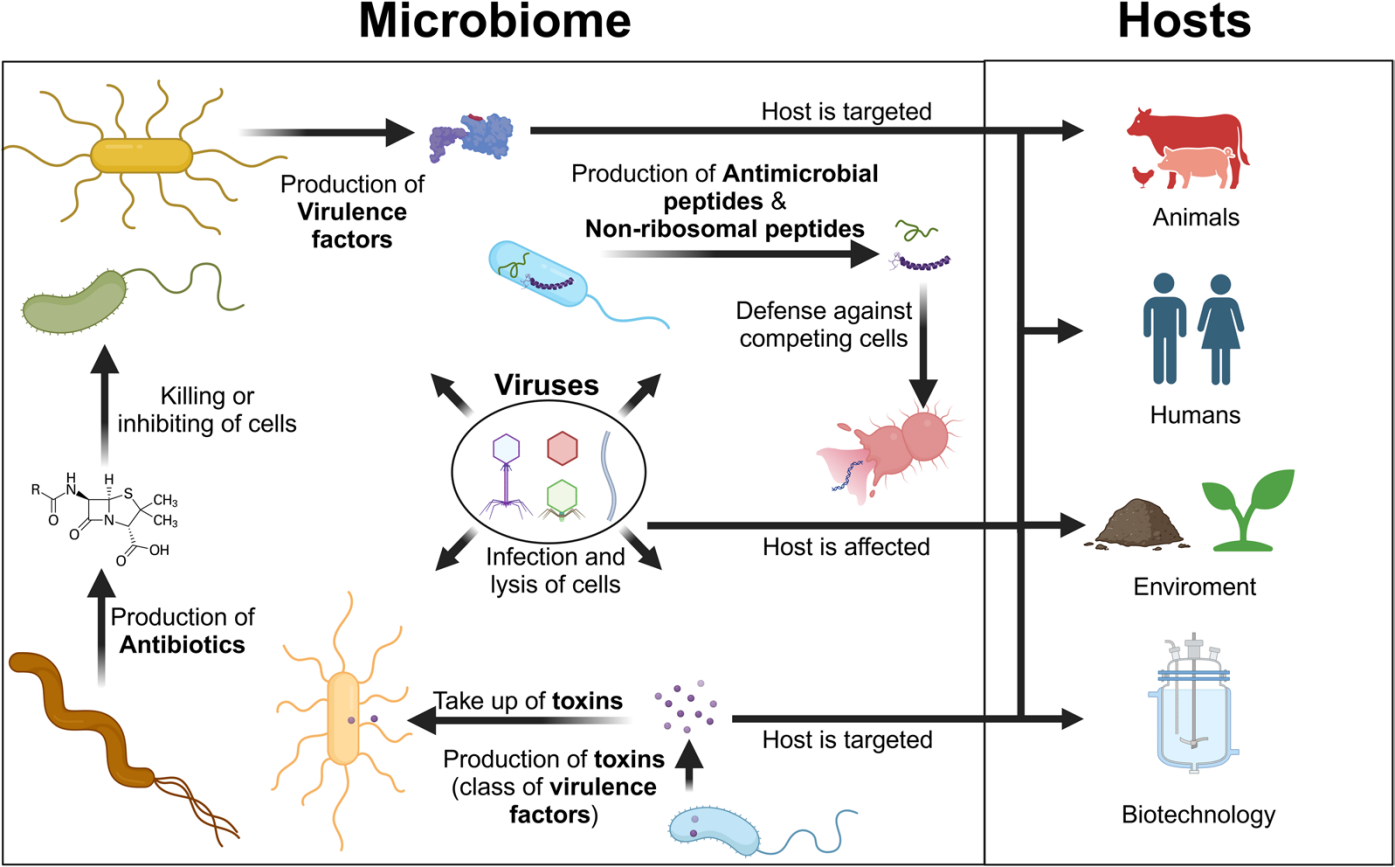
Overview of the role of microbial effectors in microbiomes and their interaction with the hosts

Microbial effectors encompass a diverse range of biomolecules that microorganisms utilize to compete with other species or modify their environment (Fig. 1, Table 1, see also Table 2 for a comprehensive overview of databases for microbial effectors). For instance, microorganisms produce virulence factors to infect hosts, evade immune defenses, and cause disease. Among these, toxins are exceptionally potent, as their highly specific enzymatic activities allow them to damage host cells and disrupt biological processes even in trace amounts. In addition, many microbial species synthesize antibiotics to inhibit the growth of competing species or eliminate them entirely. Furthermore, nearly all living organisms produce antimicrobial peptides (AMP) as a defense mechanism against bacteria, viruses, fungi, and even tumor cells. These AMPs can be classified into ribosomal peptides and NRPs. Another class of microbial effectors is viruses and phages, which function as mobile, self-replicating genetic elements. The unique advantage of metaproteomics is its ability to confirm the actual presence of these microbial effectors and elucidate the involvement of specific protein machinery in their synthesis. Additionally, metaproteomics data correlate well with the actual functional microbial activity and identify host proteins present in the sample, providing valuable insights into host responses. For a selected example of the application of metaproteomics to microbial effector research, please refer to Table 3.

**Table 1:** Overview of microbial effectors

Microbial effector	Definition	Structure	Producer	Target	Resistance
Virulence factor	Molecules or structures that enable pathogens to infect hosts, evade immune defenses, or cause disease	Enzymes, toxins, adhesins	Bacteria, Archaea, Fungi, Viruses	Hosts including animals and plants	Usually slow, but fast adaptation by immune system
Toxins	Substances produced by organisms to damage host cells, disrupt biological functions, or cause toxicity	Complex metabolites (may contain amino acids)	Bacteria, Archaea, Fungi, Viruses	Hosts including animals and plants	Usually slow, but fast adaptation by immune system
Antimicrobial peptide	Short peptides produced to defend against bacteria, viruses fungi and tumor cells	Canonical amino acids	All species	broad range against bacteria, virus, fungi or parasites	Rare
Non-ribosomal peptide	Peptides synthesized by non-ribosomal peptide synthetase (NRPS) enzymes, not ribosomes	Peptide secondary metabolites	Bacteria, Archaea, Fungi	broad range against bacteria, virus, fungi or parasites	Rare
Antibiotics	Chemical compounds that specifically inhibit bacterial growth or kill bacteria	Diverse small molecules (may contain amino acids)	Bacteria, Archaea, Fungi	Target microbial structures	Frequent
Viruses	Infectious agents consisting of nucleic acids	Nucleic acids (DNA/RNA) and proteins; sometimes lipids	Self-reproducing in hosts	All domains	Usually slow, but fast adaptation by immune system

**Table 2:** Overview of databases and tools for microbial effectors

Microbial effector		Name	Description	Source
Virulence factors	Database	VFDB	- Virulence factors of bacterial pathogens	[12]
	Tool	PATHOFact	- Prediction of AMR genes, virulence factors, toxins, and BGCs	[13]
		MetaVF	- Identification of pathobiont-carried VFGs at the species level	[14]
Toxins	Database	Toxinome	- Bacterial protein toxin	[15]
		TADB	- Bacterial types I to VIII toxin-antitoxin loci	[16]
	Tool	PathoFact	- Prediction of AMR genes, virulence factors, toxins and BGCs	[13]
Antimicrobials	Database	AntibioticDB	- Antibacterial compounds (incl. discontinued agents and drugs under pre-clinical development or in clinical trials)	[17]
		DrugBank	- Drugs, drug targets, and related pharmaceutical information	[18]
		PubChem	- Chemical information on e.g., small molecules, siRNA, miRNA, lipids or carbohydrates	[19]
		ChEMBL	- Bioactive molecules with drug-like properties	[20]
		CARD	- Antibiotic resistance ontologies with curated AMR gene sequences and resistance-conferring mutations	[21]
	Tool	antiSMASH	- Detecting and characterizing biosynthetic gene clusters (BGCs)	[22]
		ResFinder	- Identification of AMR genes in NGS-data	[23]
Antimicrobial/non-ribosomal peptides	Database	CAMP_R4_	- Natural and synthetic AMPs	[24]
		dbAMP	- Annotations on AMPs (incl. sequence information, functional activity data, or physicochemical properties)	[25]
		DBAASP	- Sequences, chemical modifications, structures, bioactivities and toxicities of AMPs	[26]
		DRAMP	- Antimicrobial, antifungal, antiviral, anticancer, antitumor, antiprotozoal, and insecticidal peptides	[27]
	Tool	Macrel	- Prediction of AMP sequences from genomes and metagenomes	[28]
		SPEQ	- Identification of high-quality, not identified LC–MS spectra	[29]
		Ensemble-AMPPred	- AMP prediction and recognition from sequence data	[30]
		Deep-AmPEP30	- Prediction of short-length (≤ 30 aa) AMP	[31]
		SBSPKSv3	- Prediction of macrocyclized structures of non-ribosomal peptide synthetase and polyketide synthase	[32]
		NRPminer	- NRP discovery from (meta)genomic and mass spectrometry datasets	[33]
		BiG-MEx	- Identification of BGC protein domains and assessment of diversity and novelty	[34]
		BiG-SCAPE	- Analysis of sequence similarity networks of biosynthetic gene clusters and gene cluster families	[35]
		NaPDos	- Assessment of secondary metabolite biosynthetic gene diversity and novelty of in organisms and environments	[36]
Bacteriophages/Archeophages	Database	PhageDive	- Experimental data (e.g., host range) and metadata (e.g., geographical origin) on bacteriophages	[37]
		Gut Phage Database (GPD)	- Non-redundant viral genomes obtained by mining human gut metagenomes and reference genomes of cultured gut bacteria	[38]
		*M*icrobe *V*ersus *P*hage (MVP)	- Phage–microbe interactions	[39]
		PhagesDB	- Interactive website for discovery, characterization, and genomics of viruses that infect Actinobacteria	[40]
		PhaLP	- Phage lytic proteins	[41]
	Tool	phageAI	- Lifecycle prediction tasks based on bacteriophage nucleotide sequences	[42]
		What the Phage	- Identification and analysis of phage sequences	[43]
		PHASTEST	- Identification, interactive visualization, and annotation of prophage sequences within bacterial genomes or plasmids	[44]
		PEPGM	- Taxonomic inference of viral proteome samples	[45]
		VirHostMatcher	- Prediction of virus-prokaryote interactions	[46]

**Table 3:** Examples for the usage of microbial effectors

Microbial effector	Use case description	Source
Virulence factors (VF)	- Identification of virulence factors in body fluids (e.g., sputum) and at marine plastic surfaces	[47, 48]
Toxins	- Assessment of metabolic changes of the gut microbiome in response to toxins	[8]
	- Verification of the expression of protein toxins and proteins involved in their biosynthesis and secretion	[49]
Antimicrobials	- Revelation of complex cellular/metabolic responses included in antimicrobial-resistance (e.g., increased production of outer membrane proteins)	[50, 51]
Bacteriophages/Archeophages	- Assessment of host-phage interactions (over time), e.g., via the host immune response	[52, 53]

### Virulence factors

Virulence factors support microorganisms in host infection and disease [54]. Many of these factors are either secreted, membrane-associated, or cytosolic proteins including toxins, adhesins, invasins, proteases, and hemolysins. Other proteins are involved in biosynthesis and secretion systems for non-protein virulence factors (i.e., capsules and endotoxins) [55]. Genes encoding virulence factors can be transmitted between or within species, facilitating the selection of pathogens of enhanced virulence and driving the emergence of new diseases [56].

The transfer of DNA and thereby virulence factors is either accomplished by transduction through phages, conjugation through pili, or by uptake of naked, extracellular DNA [57]. In particular, environmental microbiomes such as those in wastewater treatment plants are reservoirs for clinically relevant virulence genes and are important drivers for the exchange and transmission of genes [58, 59]. Therefore, monitoring the presence of virulence factors in high-risk environments is a key task of the One Health framework. While metagenomics and qPCR can identify the presence of virulence genes [60, 61], these approaches may underestimate the presence of pseudogenes and cannot reveal the actual synthesis and secretion of virulence factors [62, 63]. On the contrary, proteomics and metaproteomics methods are more suitable for exploring protein synthesis and secretion according to environmental stimuli.

### Toxins

Toxins are a subclass of virulence factors comprising diverse bioactive compounds, including proteins [64]. They can be classified based on their biological effect on the target organism and on whether they are released to target cells (exotoxins) or cell-associated (endotoxins) [65]. Protein toxins are active at very low concentrations, posing severe or even life-threatening risks to human and animal health. They can either act on the cell surface, by interfering with signal transduction, damaging the membrane, or intracellularly, where they induce cell death, cytoskeleton alteration, or blockade of exocytosis [66].

Toxins readily transfer between environments. For instance, toxins produced by cyanobacteria in an aquatic environment can accumulate in fish or seafood [67]. Mycotoxins in contaminated feed can impact the intestinal microbiome and cause hormonal changes in pigs [8] and can accumulate in chicken tissue [68], which are then consumed by humans and animals, impacting their health. Additionally, soil-derived toxins can be mobilized by water, spreading across ecosystems.

Notably, xenobiotics derived from microbial toxins are currently being explored for their use in cancer treatment [69]. Therefore, identifying toxin-producing microorganisms and detecting toxins in complex matrices (environmental samples or body fluids) is crucial for the One Health framework. Recently established databases containing information about microbial toxins and antitoxins [15], and tools for predicting toxin genes in (meta-) genomic data [61], will enable the in-depth exploration of these microbial effectors.

### Antimicrobials

Antimicrobials are capable of killing or inhibiting the growth of bacteria (antibiotics), viruses (antivirals), or fungi (antimycotics). The resistance of bacteria against antimicrobials represents a significant public health concern [1]. Antibiotics are generally classified by their molecular targets, such as the bacterial cell wall, cell membrane, essential bacterial enzymes, or protein synthesis. Several databases are available for more detailed information on antimicrobials, including AntibioticDB [17], DrugBank [18], PubChem [19], and the ChEMBL [20] databases. Additionally, databases such as CARD [21] and ResFinder [23] focus on collecting AMR genes.

Numerous studies have explored antibiotic modes of action, resistance mechanisms, and bacterial screening for antibiotic resistance. (Meta)Proteomics plays a key role in elucidating how antibiotics work and uncovering cellular mechanisms of microbial adaptations to antibiotics—i.e., resistance to antibiotics [70–72].

### Antimicrobial peptides/non-ribosomal peptides

AMPs [73] are a subgroup of antimicrobials consisting of polypeptides of 12 to 50 amino acids, produced as part of the innate immune system response in all higher eukaryotes but are also found in microorganisms. They play a key role in defending against other microbial species and may even target cancer cells, fungi, or viruses [74]. AMPs are synthesized either through ribosomal pathways, utilizing canonical amino acids, often followed by extensive post-translational modification as in the case of ribosomally synthesized and post-translationally modified peptides (RiPPs) [75, 76], or via non-ribosomal peptide synthetases (NRPS), enormous multifunctional enzymes found in bacteria, fungi but also higher eukaryotes [77].

Ribosomal AMPs were once thought to be mainly linear, cationic peptides with membrane-disrupting activity, but the discovery of new RiPP classes in the past 15 years has challenged this view. Today, several highly complex RiPPs are known, which are so heavily post-translationally modified including via additional ring systems, epimerizations, hydroxylations, acylations, and/or C- and N-methylations that they can hardly be recognized as being of ribosomal biosynthesis origin. However, the big difference to NRPS-derived peptides is that RiPPs initially rely only on the 20 canonical amino acids while in NRPS-derived peptides more than 400 different amino acids have been described. Most of these are incorporated into the peptide during the assembly-line-like mechanism, where they can also be modified by C- and N-methylation, hydroxylation, oxidation, dehydralation, heterocyclization, acylation, or formylation. The resulting peptide can also be further modified post-NRPS by glycosylation, phosphorylation, sulfation, or deacylation. Furthermore, NRPS can occur in combination with polyketide synthases (PKS), forming the so-called NRPS-PKS hybrid enzymes, based on the shared biochemical mechanism, whereby all biosynthesis intermediates are covalently bound to a peptidyl- or acyl-carrier protein or thiolation (T) domain, ensuring an efficient combination of amino acids with (further functionalized) malonyl- or acetyl-units. The resulting gamma- (elongation with one) or epsilon- (elongation with two PKS units) amino acids can add to the complexity of NRPS-derived peptides beyond what is possible through RiPPs.

The chemical diversity of NRPs and RiPPs, including various modifications, makes their identification difficult, as their D- or modified amino acids and cyclic structures confer protease resistance. Most known cases were identified due to strong antimicrobial activity or through detection of the biosynthetic gene cluster (BGC) with characteristic modifying enzymes (e.g., radical SAM), followed by heterologous production of the peptide. Identifying the classical linear AMPs is also very challenging; although they can be cleaved by proteases because of their linear structures, the presence of several cationic amino acids (i.e., lysine or arginine) residues often results in peptides too small for definitive identification. The potential benefit of metaproteomics is that it is possible to quantify the abundance of the NRP-producing enzymes even in microbial communities and thus predict their presence and structure, required for their targeted measurement. Furthermore, metaproteomics studies may include spectra of NRP and RiPPs, but these have largely been overlooked due to the absence of suitable bioinformatic workflows.

### Bacteriophages and archaeophages

Phages are viruses that selectively infect and lyse bacteria and archaea, thereby shaping microbial population dynamics [78].

They are mostly divided into two groups, temperate and virulent phages. While temperate phages can integrate their genome into the genome of bacteria (prophage), virulent phages can only replicate within the bacterium and lysate cells for the viral progeny release, which occurs thanks to the production of holin and endolysin [79].

Bacteriophages are also able to target biofilm-embedded bacteria, by degrading extracellular matrix due to phage depolymerases, and to kill persister cells [80].

Phages are the most abundant biological entities in the biosphere [81]. They have been identified in different matrices (wastewater, soil, feces). Considering the small number of sequenced phages, most phage proteins cannot be identified yet, due to the scarcity of primary sequenced data, and the functionality of most of their proteins remains unknown.

Historically phage research has extensively focused on horizontal gene transfer and transduction processes, especially for toxin genes. In this context, metaproteomics might confirm the protein expression of prophages predicted by metagenome analysis. Interest has recently increased in research on phages for their potent antibacterial repertoire, including enzyme activity against antibiotic-resistant strains. These features not only support the potential use of phages as adjuvants to antibiotics for the eradication of drug-resistant bacteria but also enable the modulation of pathogenic, commensal, and pathobiont bacteria of the microbiome, thereby impacting host physiology and the immune system within a One Health framework.

## Overview of metaproteomic workflows and requirements for the analysis of microbial effectors

**Fig. 2:**
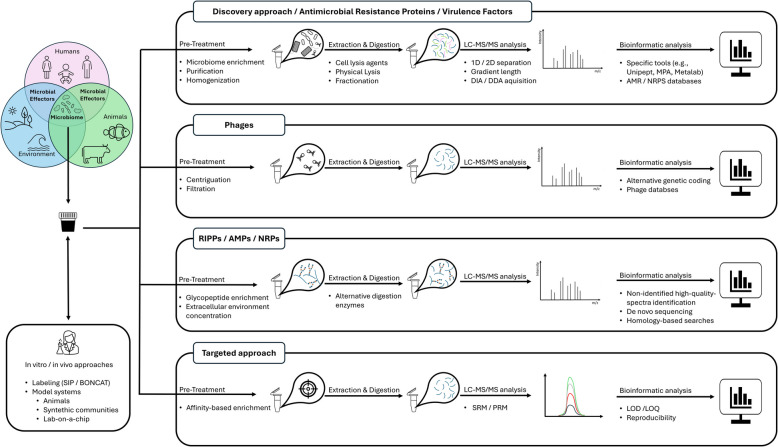
Overview of metaproteomic workflow and key aspects that must be considered for studying microbial effectors and their impact on microbiomes. Abbreviations: RIPP ribosomally synthesized and post-translationally modified peptides, NRP non-ribosomal peptides, AMP antimicrobial peptides, SRM selected reaction monitoring, PRM parallel reaction monitoring, LOD limit of detection, LOQ limit of quantification, DIA data-independent acquisition, DDA data-dependent acquisition, SIP stable isotope probing, BONCAT bioorthogonal non-canonical amino acid tagging

Metaproteomics has advanced significantly with respect to sample preparation, mass spectrometry, labeling, bioinformatics, multi-omics data integration, and model systems. These developments have facilitated the identification of microbial effectors and their impact on microbiomes. However, several challenges remain in studying microbial effectors using metaproteomics (Fig. 2). Furthermore, careful consideration must be given to Meta-data handling and experimental design [82, 83] with respect to cross-sectional or longitudinal study design, appropriately age- and gender-matched healthy controls for disease populations, robust randomization procedures, careful consideration of cage effects for animal experiments, and comprehensive power analysis. Additionally, researchers should account for potential confounding variables, implement blinding techniques where applicable, and ensure adequate sample sizes to enhance the study’s statistical validity and reproducibility.

### Sample preparation for metaproteomics and considerations for the detection of microbial effectors

The initial challenge is collecting representative biomass for metaproteomic and, if needed, metagenomic analysis [84], especially in heterogeneous samples (e.g., feces, soil). Proper preservation is also crucial if transport is required before processing. Metaproteomic sample preparation is often time-consuming due to complex extraction and purification steps, depending on the sample matrix complexity [85–91]. Standard workflows include homogenization, cell lysis, protein extraction, and proteolytic digestion, followed by mass spectrometry-based proteomic analysis. Protocols are typically adapted to microbial complexity, sample impurities, biomass content, and research goals (e.g., broad coverage, pathway analysis, or detection of extracellular enzymes). For example, water microbiomes require microbial concentration rather than extensive impurity removal.

In contrast, soil and wastewater sludge samples contain high levels of organic matter, phenolics, polymers, and inorganic compounds (e.g., minerals), necessitating specialized purification and extraction protocols. Proteins may adsorb onto solid particles like clay minerals, often in a partially reversible manner. The extracted proteome can be further fractionated to enable deeper proteome coverage and identification of low-abundant microbial effectors, either before proteolytic digestion (e.g., via gel electrophoresis) or afterward (e.g., ion exchange chromatography) [92]. A particular challenge is posed by phages, hydrolytic enzymes, or small peptides secreted into the extracellular environment, as they require efficient extraction, concentration, and purification from complex matrices, along with target molecule enrichment (e.g., antimicrobial glycopeptides) for sensitive detection. Another challenge is detecting NRPs due to their complex and diverse structures. As a result, no universal enrichment strategy exists during sample preparation. However, possible NRPs can be predicted from genomic data, enabling the development of targeted workflows for detecting specific NRP subgroups and their associated proteins [93–95].

### Mass spectrometry for the detection of microbial effectors

High-resolution tandem mass spectrometry is a key technology for generating peptide tags from partial amino acid sequences, confirming protein presence and enabling accurate quantification. Peptides obtained by trypsin digestion are typically separated by hydrophobicity via reverse-phase chromatography and introduced into the mass spectrometer through a nanospray interface. After mass determination, peptides are either isolated (in data-dependent acquisition mode, DDA) or pooled (in data-independent acquisition mode, DIA) and then fragmented. The molecular masses of the resulting fragments are then measured. The initial peptide mass data helps to narrow down potential sequence candidates, while the fragmentation patterns enable precise identification of the amino acid sequence.

Among a series of crucial parameters, those for the selection and fragmentation are paramount as they can significantly enhance the number of proteins identified and quantified. Recent advances in DIA mode have shown increased sensitivity and broader protein coverage. The recent introduction of a new generation of tandem mass spectrometers, specifically well adapted to address the complexity of metaproteomes, has significantly improved throughput and dynamic range [96, 97]. Typically, 120,000 peptides can be identified and quantified within 30 min [96]. Interestingly, 12 proteins were associated with toxins, 14 with phages 1 with virulence, and 2 with antibiotic-related function in this landscape. The dynamic range observed in this dataset enables the identification and characterizing of microorganisms comprising as little as 0.1% of the total biomass. Interestingly, NRPs can be characterized using the same experimental setup, except that specific pre-enrichment should be carried out (e.g., for glycopeptides) [98]. Once peptides are characterized, targeted proteomics approaches, such as selected reaction monitoring (SRM), can facilitate the routine, cost-effective monitoring of protein marker panels across hundreds of samples. Harnessing the full potential of cutting-edge metaproteomics represents a major breakthrough for microbiome functional analysis, marking a transformative step forward in microbiome research [99].

### Labeling approaches to study the impact of microbial effectors

Strategies to label proteins, such as protein-stable isotope probing (protein-SIP), and click chemistry approaches, such as bioorthogonal non-canonical amino acid tagging (BONCAT), are additional tools to identify and quantify the impact of microbial effectors on the metabolic activities and nutrient fluxes of microbiomes. Newly synthesized proteins in actively growing cells are detectable by incorporating labels, which cause a mass shift in the peptide spectra. Protein-SIP uses the incorporation of ^2^H, ^13^C, ^15^N, or ^18^O from respectively labeled substrates [100–102], whereas BONCAT is based on the incorporation of non-canonical amino acids such as l-homopropargylglycine (HPG) and l-azidohomoalanine (AHA) [103]. Although BONCAT is often combined with high-resolution microscopy and spectroscopy [104], the combination with mass spectrometric analyses was recently shown in studying the replication of phages during microbe-phage interactions [105], and the identification of effectors on bacterial pathogen infection [106, 107]. Thus, response mechanisms on various microbial effectors and the resulting physiological mechanisms can be identified with both methods. Bottlenecks such as the restricted use of single-labeled substrates in Protein-SIP and possible growth inhibitions by reactive substrate analogs must be considered and tested beforehand.

### Bioinformatics

Bioinformatic analysis for metaproteomics [108, 109] is challenging due to the usage of metagenomes for protein identification, redundancy from homologous proteins, and the need for comprehensive taxonomic and functional annotation. To address this, several specific tools for metaproteomics were developed [110–112], improving our understanding of how microbial species contribute to resistance mechanisms across human, animal, and environmental health domains.

As outlined above, metagenomes derived from the same or similar environments, often supplemented with protein sequences from repositories (e.g., *Homo sapiens* entries in UniProt for human microbiome studies), are typically used as databases for protein identification. These metagenomes are assembled, genes are predicted (gene calling), and frequently, metagenome-assembled genomes (MAGs) are constructed to define sample-specific taxonomic units. For microbial effectors, tools such as PATHOFact (virulence factors and AMR) [61], antiSMASH (secondary metabolite biosynthesis) [113], or Macrel (AMP prediction) [28] support the annotation of genes and related proteins (e.g., polyketide synthases or resistance genes). Furthermore, VirHostMatcher [46], phageAI [42], What the Phage [43], and PHASTEST [44] can be used to identify potential bacteriophage hosts and target structures.

A non-trivial challenge in constructing metagenome databases for metaproteomics—particularly for cross-sample comparisons—is mapping genes and MAGs across different metagenomes or combined datasets. These mappings must account for subspecies diversity, mutations, and sequence variations while keeping the database compact for reliable FDR estimation. This issue is closely tied to proteogenomics, which combines genomic and proteomic data for deeper analysis [114]. In metaproteomics, proteogenomics has demonstrated that phages can employ alternative genetic coding strategies [9]. Additionally, integrating a combined database or employing advanced tools enables a more detailed taxonomic and functional characterization of microbiomes from non-sequenced hosts [9].

Another major challenge in metaproteomics is accurately identifying non-tryptic peptides and inferring homologous proteins across diverse microbial species. Due to extensive microbial genome diversity, traditional database-driven methods struggle with incomplete or mismatched databases. Many proteins, especially those involved in resistance mechanisms, are poorly represented in existing databases. This makes homologous protein identification a bottleneck where inaccurate homologous protein matching leads to gaps in understanding microbial resistance expression.

RiPPs and NRPs can be chemically highly complex from various modifications and therefore often lack predictable structures, making them difficult to detect and identify using conventional proteomics workflows. As a result, they still need to be explored despite their crucial roles in AMR. A potential solution to identify RiPPs, NRPs, and non-tryptic peptides is to screen high-quality unidentified spectra [29] and apply de novo sequencing [9] and homology-based searches [115], expanding the search space beyond predefined peptide sequences and databases. Moreover, integrating machine learning approaches and transfer learning can help refine peptide identification, making the detection of complex resistance mechanisms more accurate [116]. Another approach for identifying NRPs or RiPPs with known BGCs involves heterologous expression or genetic manipulation of the native producer, followed by the comparison of expression, deletion, or overproduction mutants with the wild-type strain. While this facilitates compound identification, it requires genetic tools for the producer and knowledge of the biosynthetic pathway.

To better understand microbial effectors, identified proteins can be mapped to metabolic networks and used as input for modeling microbiomes to study their impact on taxonomic and functional composition [117].

### Integration of further omics methods to enhance the identification of microbial effectors

**Fig. 3:**
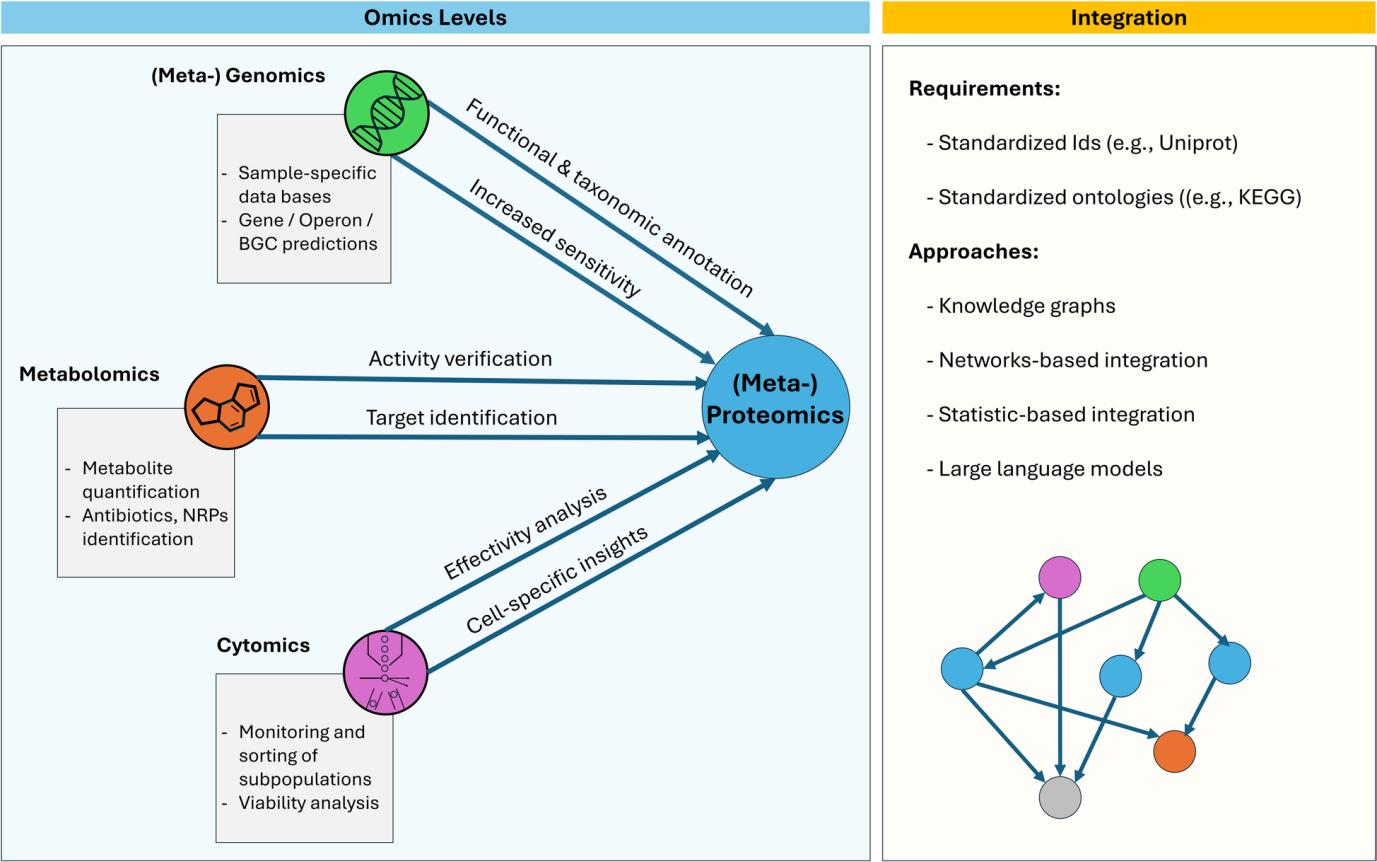
Strategies for combining metaproteomics with other omics tools

Although metaproteomics is a powerful tool for studying microbiomes and microbial effectors, its potential is greatly enhanced by integrating additional omics approaches (Fig. 3). Cytomics enables monitoring and sorting microbial subpopulations, providing insights into cell-specific behaviors. Furthermore, cytomics can measure cell viability [118], which is of great importance, e.g., to assess the response of microbiomes to antimicrobials and could even provide the potential to evaluate community structure, diversity, and metabolite exchange in response to microbial effectors [119]. As noted above, metagenomics is key for generating sample-specific databases for protein identification, while also revealing taxonomic, functional, and genomic contexts (e.g., operons, neighboring genes, mobile elements) of microbial effectors [61]. Complementarily, metabolomics—often using liquid or gas chromatography (LC, GC) for analyte prefractionation coupled with tandem mass spectrometry (MS/MS)—is a valuable tool for studying microbiomes, enabling metabolite quantification. It offers a key advantage in effector analysis by allowing broad chemical screening to identify, for example, novel antibiotics and NRPs [120, 121]. A key challenge in multi-omics research is integrating diverse data types, requiring standardized identifiers and ontologies [122, 123]. An effective approach is aggregating all data into a graph-based structure or linking it to a knowledge graph. The benefit of knowledge graphs lies in their ability to integrate heterogeneous data, apply graph algorithms [124], and facilitate connections with large language models, enabling improved data exploration and predictive analysis. Another strategy for multi-omics data integration is calculating correlation factors between the different omics features, which could be nicely visualized as co-occurrence networks [125].

### Model systems to study microbial communities for the validation of microbial effectors

Researchers require controlled experimental models incorporating both in vitro and in vivo approaches to identify and validate novel microbial effectors and their impact on microbiomes. Furthermore, controlled experiments are required to obtain ground truth datasets for method development validation [126]. Synthetic microbial communities (SynComs) represent gold-standard systems for studying microbial interactions and responses [127]. These models provide controlled environments that simulate natural microbial ecosystems, allowing for precise examination of effector molecules and their roles in community dynamics, signaling, and host interactions. SynComs offers a robust foundation for investigating microbial functions within microbiomes by enabling direct observation of cause-and-effect relationships while minimizing confounding variables. SynComs also enhances metaproteomic research by using annotated genomes for each community member, which improves protein identification accuracy. This genomic information enables detailed insights into strain-level interactions, often unachievable in natural microbiomes due to unknown protein sequences. Additionally, SynComs allow for the study of low-abundance species, such as keystone taxa [128, 129], which play essential roles within the microbiome but are often undetectable in conventional metaproteomic analyses. In vivo mouse models replicate human physiological responses for host-relevant microbial studies, providing insights into microbial-host interactions and discovering microbial effectors pertinent to disease. Emerging “lab-on-a-chip” microfluidic platforms complement, these models by allowing precise control and monitoring of microbial communities in high-throughput formats, enhancing our understanding of microbial dynamics under controlled conditions [130]. Fermentation systems simulate gastrointestinal conditions, supporting long-term studies of microbial fermentation and gut ecology [131, 132]. Of particular interest for the fermentation are systems that enable co-cultures of microorganisms and human cells, to ascertain the effect of different microbiota-expressed effectors on human cells to ascertain the effect of different microbiota-expressed effectors on the human cells [133, 134].

Together, these models offer a comprehensive toolkit for assessing microbial effects on host health, advancing our understanding of microbial communities in health and disease.

### Challenges and limitations of metaproteomics

While previous sections have addressed the individual limitations and challenges of metaproteomics, we would like to highlight these points more explicitly [109, 135, 136], particularly in the context of clinical applications. Although significant progress has been made in recent years—such as the development of combined multi-omics workflows [137], analysis times of under 24 h [138], advanced mass spectrometry methods for comprehensive microbiome characterization [139], and improvements in standardization [126, 140]—metaproteomics has yet to be established in routine clinical practice. The primary limitation is that no single metaproteomics workflow has been certified according to international standards for clinical use, such as ISO 13485 (quality management for medical devices) and ISO 15189 (laboratory quality and safety). Overcoming this limitation requires substantial resources, which often exceed the budgets of individual research groups, as well as identifying a valuable use case for clinical application.

In addition to this major hurdle, there are several smaller challenges in metaproteomics that require further refinement. First, metaproteomics remains susceptible to biases in sample preparation and needs further improvements in quantification accuracy. Additionally, the resolution of current MS devices is still insufficient to fully capture the microbiome’s complexity or to analyze it at single-cell resolution [141]. Reducing the high investment costs associated with mass spectrometers, maintenance, and specialized laboratory infrastructure would be advantageous for wider clinical adoption.

Another significant challenge lies in the bioinformatic analysis of metaproteomics data. This process is hindered by the lack of appropriate routine protein search databases, difficulties in calculating the FDR, and challenges in identifying medically relevant post-translationally modified peptides.

## Application of metaproteomics for studying microbial effectors and microbiomes in the One Health framework

To evaluate the potential for identifying and studying microbial effectors in the One Health framework, we assessed the current usage of microbiomes in the clinical (Chapter 4.1) and non-clinical context (Chapter 4.2).

### Usage of microbiomes in the clinical context and potential for metaproteomics and microbial effectors

**Fig. 4:**
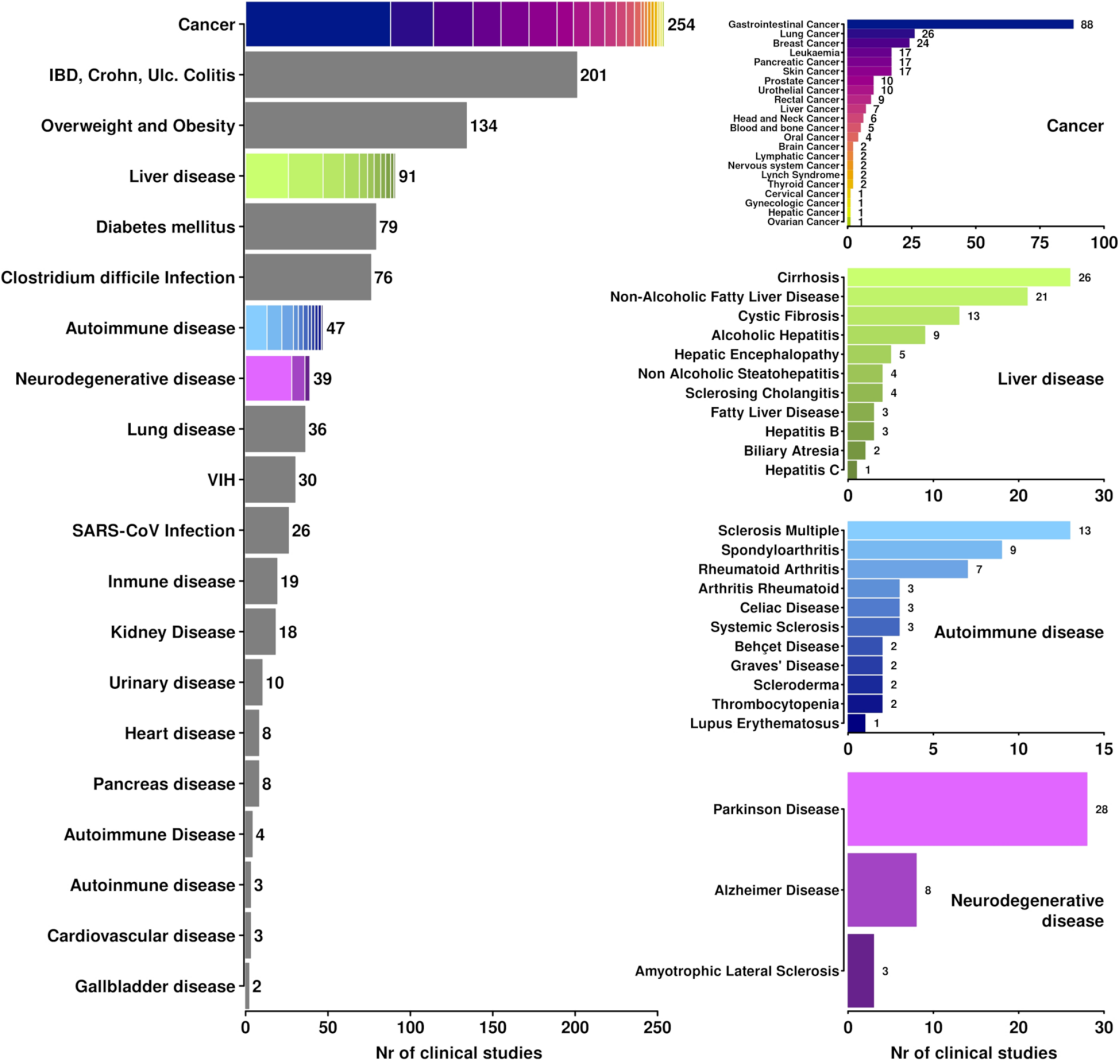
Summary of over 2400 clinical studies exploring the microbiome’s role in health, distributed by its relation to 70 diseases. Cases where multiple types of cancers or diseases are studied are detailed on the right. The figure was created using R programming language v.4.4.1, with core-base functions and in-house scripts

Currently, over 2400 clinical studies are investigating the microbiome’s relationship with various health factors (https://clinicaltrials.gov/). Of these, over 1000 focus on the microbiome’s role in 70 diseases, including autoimmune disorders, cancer, cardiovascular, digestive, and metabolic diseases (Fig. 4). These clinical studies reflect the growing recognition of the microbiome as a key factor influencing disease diagnosis, prognosis, and treatment response.

The importance of the microbiome extends beyond the human microbiome and health to encompass the interconnected animal and environmental dimensions of the One Health framework. Microbial communities in animals and the environment actively shape human microbiome composition and functionality. Through direct exposure, shared ecosystems, and environmental reservoirs, these interactions influence the microbiome’s clinical impact. For instance, zoonotic pathogens, or AMR genes are often mediated by microbial exchanges between humans, animals, and their habitats, demonstrating the profound interplay within these domains.

The high interest in the microbiome is particularly pertinent in diseases where immune and inflammatory mechanisms play a central role, as the microbiome may modulate both immune-suppressive and stimulatory pathways [142]. Furthermore, microbiome-host interactions extend beyond individual organs vital for maintaining homeostasis and influencing disease progression [143]. Therefore, the interrelationship between microbial communities colonizing different human surfaces provides the advantage of collecting highly informative profiles from more accessible microbiome samples in clinical contexts where pathology mainly affects less accessible organs.

Although different diseases are associated with distinct microbiome-host interactions, key areas of investigation remain. These include understanding the microbiome’s treatment response, metabolic consequences, and underlying molecular pathways and identifying microbiome components that enhance clinical status. To illustrate the need for microbiome studies and the microbial effectors, we present the following four clinical use cases:

(i)) Recent studies have shown that microbial proteins, which accumulate under specific conditions, as well as their sequence diversity, structural features, and post-translational modifications (PTMs) like acetylations, deaminations, hydroxylations, methylations, nitrosylations, oxidations, and phosphorylations, are critical for priming immune cells effectively [117]. Understanding these variations in proteins, whose nature can be revealed through metaproteome analysis, combined with advanced computational methods such as protein structure prediction [144] and all-atom molecular dynamics (MD) simulations [145], provides insights into the role of microbial proteins in immune regulation. This insight could potentially guide the development of targeted therapeutic strategies.
(ii)) Cancer and infectious diseases, including HIV, underscore the importance of identifying specific microbiome-derived proteins that can boost immune function and mitigate inflammation while simultaneously managing antibiotic resistance in frequently hospitalized individuals [146]. Such patients often require repeated antibiotic cycles, which further complicate treatment by promoting resistance.
(iii)) Furthermore, understanding disease requires a more holistic view. For example, *Helicobacter pylor*i was, until recently, considered the strongest risk factor for the development of gastric cancer, which is the fifth most common cancer worldwide. However, recent advances in metagenomics and metaproteomics techniques demonstrated changes in the complete microbiome during gastric carcinogenesis rather than that of single microbes. Hypochlorhydria, a state of low hydrochloric acid levels that affect the stomach’s ability to digest and absorb proteins, induces changes in the complete microbiome (reducing diversity and abundance of commensal bacteria and promoting overgrowth of pathogenic and carcinogenic species) that might have a direct link with gastric cancer [147]. This is further enhanced by the prolonged use of proton pump inhibitors, which are widely used medications [148]. Thus, the risk of cancer could be identified by characterizing microbiome alterations in patients’ gastric juices and/or feces. Monitoring microbial alterations could also help physicians and healthcare professionals assess the risks and benefits of using medications such as proton pump inhibitors, monitor medical care protocols, and optimize treatments for high-risk patients.
(iv)) Preclinical and clinical trials suggest that the alterations in the gut microbiome are also linked with toxicities induced by chemotherapies [149] and immunotherapies [150]. It has also been suggested that modulation of the gut microbiome before and during chemotherapy in cancer patients could reduce the occurrence of adverse events and improve the effectiveness of treatments [151]. Recent studies also suggested that the gut microbiome, available via fecal material, constitutes a promising source of biomarkers to predict and monitor treatment outcomes and potentially related adverse events [152]. Furthermore, tongue swab metaproteomics has, for instance, enhanced our understanding of the mechanism behind specific tongue coating formation and its potential role as an indicator of gastric cancer [153].

In sum to these examples, clinical needs for microbiome research include (i) accurate and timely diagnosis of microbiome functional alterations, (ii) monitoring the disturbances in microbial communities and their components (genes, transcripts, proteins, metabolites) triggered by clinical protocols, (iii) evidence-based therapy to modulate the microbiome and regain its homeostasis, and (iv) the identification of novel microbial effectors for targeted microbiome management. Metaproteomics can provide solutions to these critical domains by profiling how clinical traits shape the microbiome, identifying microbial effectors involved in variations of the microbial community structure and functions, and monitoring the outcome of experimental clinical protocols based on microbial modulators, including AMPs and phages.

In the context of microbial effectors, metaproteomics holds great potential for (i) investigating the therapeutic use of phages as antibacterial agents across various clinical conditions, (ii) monitoring the functional dynamics of their bactericidal activity, and (iii) assessing therapy responses by analyzing correlations between bacterial and host protein profiles.

### Non-clinical microbiomes and microbial effectors in the One Health framework

The connection between microbiomes across environmental, agricultural, and biotechnological domains and the One Health framework extends beyond serving as a reservoir of novel microbial effectors for human therapeutics. Many microbial effectors developed for human use can also benefit pet and livestock health.

In crop and horticulture science, microbial antimicrobials such as cyclic lipopeptides produced by *Pseudomonas* strains can act as natural insecticides, effectively targeting insect larvae. Phages may be employed to combat plant pathogens like *Pectobacterium atrosepticum*, which causes potato soft rot [154], while seed coatings with antimicrobial agents offer protective benefits [155]. Additionally, antimicrobials can enhance food safety by reducing microbial contamination in production and food supply chains [154, 156].

In environmental management, cyanophages could be harnessed to mitigate harmful algal blooms, thus safeguarding aquatic ecosystems like oceans, seas, and lakes [157]. Meanwhile, antibiotics and other antimicrobials might stimulate the growth of contaminant-degrading microbes in nutrient-limited environments, such as certain groundwater systems, aiding in bioremediation [158].

Within biotechnological applications, phages offer a targeted approach to controlling filamentous bacteria, including *Microthrix parvicella* and *Nocardia* species, which cause foaming issues in wastewater treatment plants [159]. Phages also have emerging applications as structural components in nanomaterials, presenting exciting opportunities in materials science [160].

While microbial effectors offer significant potential, it is crucial to consider potential unintended impacts on microbiomes, such as effects on non-target species and the development of resistance mechanisms. Additionally, stressors—including those from human activities—can accelerate the release of phages within microbiomes, leading to self-amplifying cycles and other stress responses. For instance, exposure to pesticides has been shown to increase bacterial antibiotic resistance by activating efflux pumps, reducing outer membrane permeability, and inducing gene mutations [161].

## Future potential of microbial effectors and metaproteomics in the One Health framework

Reverse to the spread of pathogenic species across different hosts (zoonoses), using microbial effectors from diverse environments holds transformative potential for treating diseases and controlling microbiomes in biotechnological systems. Just as the “golden age” of antibiotic discovery opened new frontiers in medicine, broader screening of microbial effectors now offers the potential to treat pathogen-associated diseases. One advantage of many microbial effectors is their ability to target specific microorganisms, allowing for more precise treatment options and enhanced microbiome control. However, this specificity also increases the risk of resistance mechanisms, such as escape mutations, which require continuous adaptation of microbial effectors to maintain efficacy.

In this context, metaproteomics plays a crucial role in advancing microbial effector research through two key contributions: (i) metaproteomics enables the comprehensive characterization of microbiomes and their expressed proteins, leading to the identification of novel microbial effectors and their mode of action.

Therefore, it should be applied across various environments to maximize the number of novel microbial effectors identified. Environments under selective pressure—such as reptile saliva, amphibian skin, hospital wastewater, and livestock enclosures—are particularly interesting for discovering novel compounds. (ii) Metaproteomics allows for the analysis of expressed proteins and phages, and it is an ideal tool for examining the effects of microbial effectors.

Beyond microbiome monitoring, metaproteomics data can inform microbial abundance estimates, which are essential for developing control algorithms to manage microbiomes effectively [117]. Using these algorithms, control variables such as nutrient supply, process parameters (e.g., temperature), or the introduction of microbial effectors can be adjusted to achieve the desired microbiome functionality. This concept of microbiome management is indeed analogous to animal gastrointestinal tracts regulating their microbiomes. Closing the gap to the One Health framework, the most comprehensively studied system for microbiome management is the human gut, whose control mechanism (e.g., AMPs) could also be applied to manage microbiomes in environment and biotechnological applications.

Another application of metaproteomics lies in “pandemic preparedness” within a One Health framework. For example, tracking pathogen concentrations in wastewater treatment plants, as seen with COVID-19, enables early detection of disease outbreaks before hospitals or government agencies identify them. Sequencing methods with low detection limits (enabled by gene amplification) are primarily used for such monitoring. As demonstrated with the selective enrichment of COVID-19 peptides using advanced mass spectrometry (LC–MS/MS), proteomic methods could also play a valuable role in pandemic preparedness, offering timely and reliable pathogen monitoring enabling the monitoring of the actual expression of the pathogens [162]. To accomplish this, tools have been developed to identify viruses from LC–MS/MS-based peptide identifications [45]. As a result, the holistic tracking of wastewater through metaproteomics emerges as a powerful approach for detecting emerging pathogens and microbiome dysbioses associated with human diseases.

## Concluding remarks

In summary, metaproteomics holds significant potential for elucidating microbial effectors and their impact on microbiomes and for monitoring the molecular processes underlying the One Health framework. However, integrating information across various levels is essential to holistically understand microbiomes and One Health. This integration requires interdisciplinary collaboration among experts from diverse microbiome-related fields and the complementary use of different methods. Metaproteomics uniquely enables the quantification of expressed proteins, including their modifications. This information allows for assessing the microbiome’s functional expression (e.g., phages, AMPs) and catalytic potential. However, to capture the complete picture of the microbiome, metagenomics is essential for monitoring genetic potential and providing the necessary databases for metaproteomics. Additionally, combining metabolomics with metaproteomics data offers insights into the actual activity of the microbiome. Beyond omics-level analyses, further mechanistic studies are crucial to validate the identified findings and mechanisms and develop mathematical models to control and optimize microbiomes.

## Data Availability

No datasets were generated or analysed during the current study.

## Abbreviations

AHA: L-azidohomoalanine
AMP: Antimicrobial peptide
AMR: Antimicrobial resistance
BGC: Biosynthetic gene cluster
BONCAT: Bioorthogonal non-canonical amino acid tagging
DDA: Data-dependent acquisition
DIA: Data-independent acquisition
FDR: False discovery rate
HPG: L-homopropargylglycine
LC: Liquid chromatography
LOD: Limit of detection
LOQ: Limit of quantification
MAG: Metagenome-assembled genome
MD: Molecular dynamics
MS/MS: Tandem mass spectrometry
NRP: Non-ribosomal peptide
NRPS: Non-ribosomal peptide synthetases
ompA: Outer membrane proteins
PKS: Polyketide synthases
PRM: Parallel reaction monitoring
PTMs: Post-translational modifications
RIPP: Ribosomally synthesized and post-translationally modified peptides
Protein-SIP: Protein-stable isotope probing
SRM: Selected reaction monitoring
SynComs: Synthetic microbial communities
T: Thiolation
